# Integration of Multi-Omics in AI-Based Models for Predictive Oncology

**DOI:** 10.3390/genes17101231

**Published:** 2026-10-04

**Authors:** Arnaud Nativel, Sarah Dandou, Florian Favier, Candice Lesage, Alain Mangé, Julien Aligon, Peter J. Coopman, Romain M. Larive

**Affiliations:** 1 IRCM, Université de Montpellier, ICM, INSERM, F-34298 Montpellier, France; arnaud.nativel@umontpellier.fr (A.N.);; 2 Department of Dermatology, Saint Eloi Hospital, Montpellier University Hospital, 34090 Montpellier, France; 3 Institut du Cancer de Montpellier, Département D’Oncologie Médicale, F-34298 Montpellier, France; 4 Université Toulouse Capitole, IRIT, (CNRS/UMR 5505), 31042 Toulouse, France; 5 IRCM, Université de Montpellier, ICM, INSERM, CNRS, F-34298 Montpellier, France; peter.coopman@inserm.fr

**Keywords:** hybrid AI, multi-omics, oncology, system biology, machine learning

## Abstract

Cancer heterogeneity and the emergence of innovative therapeutic alternatives have made the development of tools to help in clinical decision making even more necessary. The recent advances in the field of artificial-intelligence-based predictive models make them promising allies due to their ability to integrate vast amounts of descriptors and data, notably omic data, for grasping cancer complexity. However, it is difficult to present a complete picture of this field due to the increasing number of models and the many possible applications. The aim of this review was to offer an overview of omic-based predictive models in oncology through the prism of three important concepts: the objective, the data, and the model. We show that the consideration of these three aspects could help to assess the feasibility, validity and usability of a model and to guide its construction. Lastly, we highlight that taking into account the underlying technical, physiological and biological phenomena inherent to omic data at each step of the project may improve the model predictive capability and its explainability.

## 1. Introduction

Decades of basic research and clinical practice have progressively unraveled cancer complexity and led to considerable improvement in its management. An important milestone has been the discovery of tumor-driving genes (i.e., oncogenes) and the development of targeted therapies that can increase patient survival [1]. However, targeted therapies and the more recent immunotherapies demonstrate variable efficacy depending on the patient, tumor type and tumor stage. Therefore, new theoretical, diagnostic and therapeutic tools are needed [2,3,4]. This has also led to the emergence of precision oncology [5], the aim of which is to better characterize individual tumors in order to propose a tailored treatment, instead of the traditional “one-size-fits-all” approach. This has been facilitated by the development of new analytical technologies, such as Next-Generation Sequencing [6,7] and liquid biopsy [8], that have also led to the generation of an even larger amount of information on a tumor, complementing the existing methods that are mainly imaging-based [9].

Robust data exploitation methods are the second cornerstone in precision oncology. They rely on computational tools and models to disentangle the complex interactions between biomarkers and clinical outcomes of interest [10,11]. The past ten years have witnessed an increase in computational power and the emergence of new methodological approaches. Artificial intelligence (AI) and machine learning (ML) represent major opportunities to advance our understanding of cancer mechanisms through improved data integration and to enhance the predicting capability of precision oncology [12].

In biology and also in oncology, predictive models have been constructed and used for a long time [13]. However, the integration of multi-omics data brings new challenges due to their high dimensionality and their complex underlying interdependencies [14]. Many reviews already offer an overview of multi-omics integration in precision oncology [12,15,16,17,18]. Lipkova and coworkers tackled the notion of multimodal data integration (omics data in addition to clinical and imaging data) for artificial intelligence in oncology, highlighting two mains concepts: the type of used AI methods between supervised, weekly supervised and unsupervised, related with the expected outcome of the multimodal data exploitation, and the type of multimodal data integration methods between early, intermediate and late fusion, depending on data type, as well as the expected outcome [15]. Wei and coworkers highlighted the same notion summed into a processing pipeline, composed of four steps: data preprocessing, integration, ML/DL modeling, and validation. While the fusion method is hidden in the integration step, with two substeps, feature selection and feature extraction, they similarly mentioned the distinction between supervised and unsupervised methods. Interestingly, they elaborated the importance of validation to assess the risk of bias and potential usefulness of models with distinction between retrospective and prospective validations [16].

More recently, Yadav and coworkers presented a similar five-step workflow to build a model: data preprocessing, feature engineering, model training, biological interpretation, and clinical application [12]. They tackled the distinction between the integration of individual omics and multi-omics integration in a model, highlighting the specific challenges and methods to ensure robust exploitations of these omics in the different contexts. One can note the addition of a biological interpretation step. Liu and coworkers reviewed the most recent application of multi-omics in precision oncology, and advocated for a better integration of these models into the ecosystem, through the integration of digital twins [16]. These different reviews highlight the continuous work that has been done by the community to provide a more robust and standardized building process of models for predicting purposes in precision oncology.

From a theoretical perspective, prediction assumes the existence of conserved relations (correlation or potentially causality) between variables [19], and relies on a model, a representation of the relation, that indicates how variations in one variable are transmitted to other elements [20]. While ML models can detect and discover these relations through the data, some of them may already be known and we believe that the integration of this prior knowledge may help to tackle certain challenges mentioned above. Thapa and coworkers have published a detailed review on how to integrate prior knowledge at different steps of the deep learning model-building pipeline, tackling issues like representation of prior knowledge in graphs [18].

With this paper, we would like to pursue efforts toward the consideration and integration of prior knowledge, not only in the model, but also in the construction process of the model: objective, data and model.

We think that the concepts should guide the design/development of predictive models in precision oncology to overcome these new challenges. In the first section, we examined the types of predictive objectives that can be derived from multi-omics and how these objectives can be shaped by the data. In the second section, we showed how objectives and data can guide the choice of model.

## 2. Prediction in Oncology

As the myriad of elements that compose a cancer are progressively unraveled, it has become necessary to associate them with the deregulation of homeostatic biological functions observed in this disease. Consequently, the concept of cancer hallmarks was born [21]. It integrates the macro-level phenomena that support tumor growth (e.g., self-sufficient growth signaling, apoptosis escape) and the cancer adaptation to its environment (e.g., immune escape, angiogenesis). Each tumor presents a unique combination of these aspects, and their characterization might help to select the most adapted therapy [22]. For example, immune checkpoint inhibitors have demonstrated higher efficacy in “hot” tumors characterized by high immune infiltration and high mutational burden [23].

**Figure 1:**
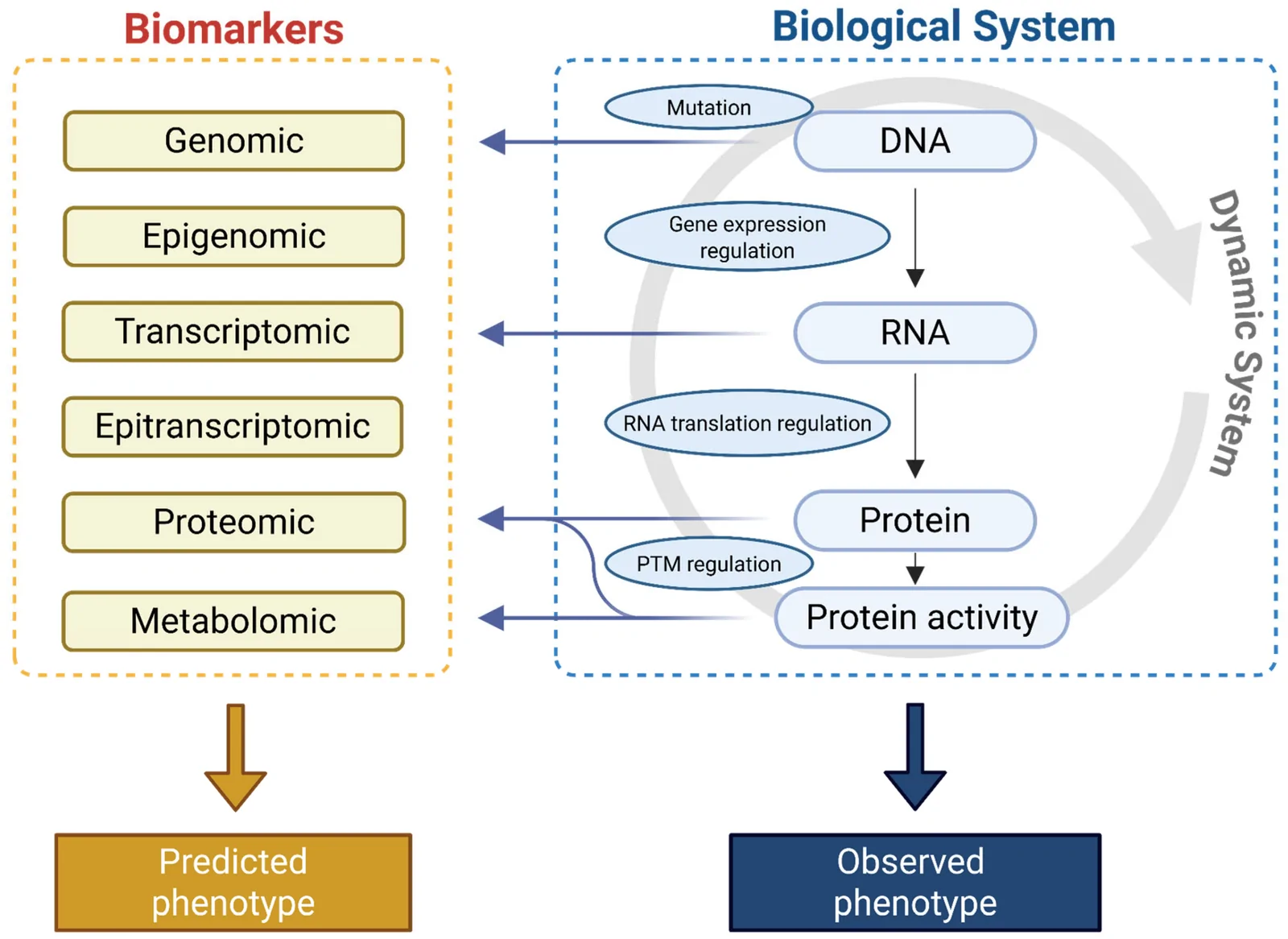
Representation of the information transfer from a dynamic biological system to the identification of biomarkers (red) for phenotype prediction (plain orange frame). Information regarding the observed phenotype (plain blue frame) is carried by different molecular actors that undergo several physiological and pathophysiological modifications regulating their activity (blue dashed frame). Multi-omics (brown dashed frame) allow identifying biomarkers to monitor the state of the biological system at its different levels. PTM: post-translational modification.

Most cancer hallmarks result from modifications of the cellular machinery [22]. In biological systems, molecules can be divided into different families in accordance with their biological function and can be analyzed individually or simultaneously (Figure 1) [24]. The DNA–RNA–protein axis forms the backbone of this machinery, which is regulated by numerous signals, from external stimuli to internal feedback loops. Cancer is characterized by deregulation of this axis at different levels (e.g., gene mutations, pro-tumoral micro-environment changes) that then translates into the macrophenomena that compose the observed phenotype. As omics technologies are used to quantify or characterize the molecules that compose the different layers of the machinery, they are a major source of biomarkers to characterize and predict these phenotypes. Thus, the adoption of robust omics data integration and interpretation techniques is a promising step toward successful predictive precision oncology [25,26].

The great dimensionality of omics data and the underlying biological mechanisms they reflect are both challenges and advantages that must be taken into account when defining a predictive objective. As highlighted previously, prediction relies on the concept of the model. From a conceptual point of view, a computational model is represented as a function that transforms inputs (including omics data) into outputs (the prediction). From this representation, one can highlight two aspects: the shape of the function that performs the transformation of the inputs into outputs, and the input context for which this transformation is valid.

Models employing “omics data” regroup very different data situations: single omic modality, several omic modalities, multimodal (omics and clinical and/or imaging data), bulk, single cell, and spatial single cell. While each of these scenarios presents specificities, we believe that many encountered challenges and methods are applicable to the other omics data situations. Hence, this review will rather focus on the definition of these challenges and methods, indistinctively of the data omics situations that they originate from.

### 2.1. Prediction Objectives

**Figure 2:**
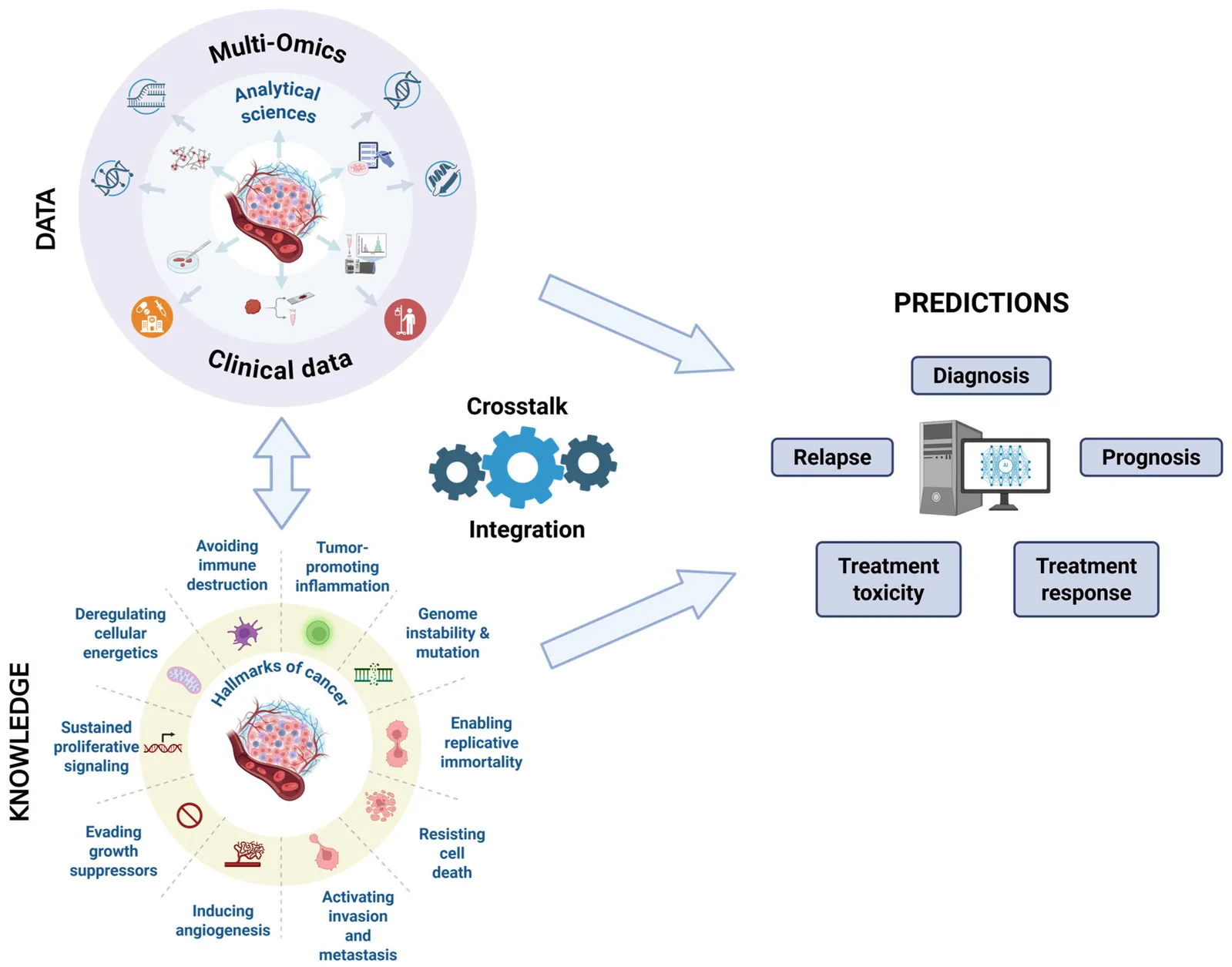
Integrating multi-omics data with the knowledge on cancer pathophysiological phenomena (hallmarks) could allow more robust and accurate predictive oncology. While knowledge and data often tend to be separated, there exists a continuous cycle between them: data, knowledge, hypothesis, experiment, data, etc. Besides prediction, either AI- or mechanistic-based models can help to structure the information at every step of this cycle. Inspired by Hanahan et al. [22].

To develop a successful prediction model, first, one must question the relevance of the link made between inputs and outputs [27]. In the context of predictive oncology, omics data have a highly relevant role as predictive variables in the model. However, their contribution may vary depending on the prediction objective, and the need to consider other relevant factors. As proposed in Figure 2, cancer hallmarks are appropriate intermediaries between the omics data and the predicted outcomes of interest that could help to understand and ameliorate the contribution of individual omics variables. The following section describes the different types of objectives of precision oncology for which omics have been used and discusses the relevance of omics data.

#### 2.1.1. Diagnosis

In oncology, diagnosis can be divided into three categories: cancer screening, cancer detection, and cancer profiling [28]. Nowadays, imaging and clinical data are the principal source of information used in AI applications [28]. Due to their nature, omics technologies also are promising sources of data, especially for cancer profiling and for precisely characterizing the tumor behavior.

One of the potential applications of multi-omics is the prediction of cancer subtypes. Clustering methods are used to group patients based on similarities at the level of multi-omics data. They may help to discover specific cancer subtypes in an unsupervised way [29,30], or more interestingly by integrating some knowledge through, for example, the notion of pathway activity inference [31,32]. In some cases, these cancer subtypes may uncover clinically relevant molecular behaviors [33,34].

#### 2.1.2. Prognosis

Prognosis corresponds to the expected cancer course (from relapse to death). From a mechanistic point of view, some cancer features (e.g., ability to invade the host tissues or induce distant metastasis) should influence the patient’s prognosis [35]. As such aspects are linked to the tumor molecular profile, accurate exploitation of omics data could provide valuable information for prognosis prediction. For instance, in a study with patients harboring resectable gastric cancer, the integration of single omics data permits accurate prognosis prediction. In the same study, consideration of multi-omics data even improved prediction accuracy [36]. Another study in the same cancer type showed that a specific signature derived from multi-omics data, centered around programmed cell death, is associated with substantial patient prognosis power [34]. Similarly, multi-omics data integration allowed identifying a signature based on mitochondrial-related genes in lung cancer that displays with an accurate prognosis prediction capability [37]. The prediction of metastasis from multi-omics data has been reported in various other cancer types [38,39].

#### 2.1.3. Treatment Response

Prognosis is closely related to the clinical practice and available treatments. The prediction of treatment response is one of the key domains of precision oncology where multi-omics data could bring benefits. The information provided by omics data on the molecular state of biological systems, which interferes with the mechanism of action of treatments, could allow the accurate prediction of treatment efficacy (predictive factor). Drug efficacy is evaluated at different steps during drug development, in different contexts and with different issues at stake.

Initially, the potential efficacy is evaluated in vitro in tumor cell lines. Although the representativity of these biological models compared to what happens in real life is more and more questioned [40], in vitro cell models still provide valuable information on the interaction between a drug and specific cellular phenotypes. The exploitation of multi-omics data through AI-based models could increase their usefulness. For instance, an ML model that predicts treatment efficacy based on gene expression in different tumor cell lines revealed several potentially novel drug–gene associations [41]. This issue has also been tackled by other teams using various methods and techniques [42].

Then, efficacy is evaluated using in vivo preclinical models. Few models exist to predict the relevance of mouse preclinical experiments.

Conversely, several AI-based models have been built to predict treatment efficacy in patients from omics data [43,44]. In the setting of an in vivo evaluation, we may expect a different impact of specific omics data from those in vitro, due to additional considerations like pharmacokinetics or interaction with the immune system, phenomena that are not impactful in in vitro assay [45,46]. We previously demonstrated that in a retrospective study on patients with advanced melanoma, the integration of MAPK-centered omics data increases the prediction accuracy of their response to therapies targeting proteins of this pathway, compared with the prediction based only on clinical data [47]. Other aspects of precision medicine, such as the prediction of patient relapse and drug toxicity, could also benefit from the integration of omics data in AI-based machine learning models.

### 2.2. Robustness and Validity of a Model

The robustness of a model corresponds to the conservation of a good predictive capacity despite variations in the input values [48]. The ensemble of the inputs on which the model predictions are deemed satisfying is called the “validity domain”. By construction, the data used for model training belong to it. A model holds predictive interest when its validity domain does not contain only training samples [49]. Validation methods help to assess the model robustness by evaluating the domain extension and depend on the model objectives and on the available data [48,49]. This is related to the concepts of interpolation and extrapolation in the field of modeling [50].

In models that represent building energy systems, Rätz and coworkers highlighted two uncertainty types that could hinder the model validity extension: aleatoric uncertainty (noise) and epistemic uncertainty (lack of knowledge) [51]. We propose that this distinction could be applied to models in predictive oncology based on omics data. “Epistemic uncertainty” tackles the question of generalizability of model behavior when considering knowledgeable shifts from the initial model training domain (e.g., mutation of a specific gene, or presence of a perturbation). “Aleatoric uncertainty” corresponds to the variations that derive from biological noise or analytical methods and that do not hold information regarding the outcome to be predicted and that the model should be able to mitigate.

#### 2.2.1. Epistemic Uncertainty

The handling of epistemic uncertainty of inputs has to be integrated thoughtfully during model construction and training. The use of training data that sufficiently represent the domain and cover input variations is the most widely employed strategy in ML. We call this the “representativity of the training data”. A good representativity of the training data transforms the prediction problem into an interpolation one. The standard technique to evaluate it is to divide samples between a training set and test set [49]. The well-known cross-validation methodology allows the global characterization of this dataset division, overcoming the problem of choosing an arbitrary representative test dataset in the context of the high dimensionality of multi-omics in predictive oncology [47].

Of note, the evaluation of the model predictive capability can benefit from the arbitrary selection of test data. In SCSBrC DREAM challenges, participants were invited to build an AI model to predict the protein activity of different cell lines in response to perturbations, and this with different levels of information [52]. A training dataset was provided to train the models. Then, their performance was evaluated using a test dataset known only by the organizers. This is one of the ways to ensure that there is no bias or leakage between training and test datasets.

Emergence and expansion of public databases, like TCGA, CCLE, GDSC or CPTAC, regrouping omics and clinical information of cancer biological samples, from in vitro to in vivo, represent powerful leverages to improve domain representativity of datasets used to train predictive models and thus ameliorate their predictive power. They may however suffer from some limitations like retrospective design, incomplete treatment annotation, limited diversity, small event counts, and assay heterogeneity that must be carefully considered when integrating them in a model training pipeline.

Besides the representativity of the training data, a second aspect for predicting variations in the inputs is their integration in the model, which relates to the concept of extrapolation. In ML models, this mainly depends on the chosen model and how it is factored in by the training. This can range from simple dependency in the case of linear regression models to more complex relationships [53]. Recent methods in the emerging field of Explainable Artificial Intelligence (XAI) provide tools that can be used in the context of omics-based prediction oncology to better understand these relationships [54,55].

One of the SCSBrC DREAM challenges was to predict the impact of a perturbation that has never been seen in the training data. Interestingly, “participants were encouraged to use prior knowledge for this task” [52]. In situations where training data do not completely cover the domain on which the prediction is performed, consideration of existing knowledge or of plausible hypotheses may guide the integration of inputs in the predictive model. For instance, Chen and coworkers built a deep learning model (scDeal) that they partially trained on bulk cell line data to predict the cancer drug response at the single-cell level based on single-cell RNA-sequencing data [56]. Dose exploration is another problem where the specific integration of one input (the drug dose) makes sense. Koukouli and coworkers [57] built a dose-varying regression model to predict in vitro the dose–response for each cancer cell line from their gene expression data inputs.

#### 2.2.2. Aleatoric Uncertainty

Challenges remain for the adoption of omics-based predictive AI models in clinical settings [58], and as stated earlier Yadav and coworkers offer a comprehensive overview on these aspects [12]. Models that perform well in their training and test environments may face other difficulties when applied in real-world situations, due to the inherent variability of biological systems and analytical methods to extract omics information [59], as well as tissue, cost, or workflow constraints. This leads to common issues, such as batch effect [60] and missing data [12], that must be taken into account to obtain robust prediction.

Methods to correct batch effects have been available for some time. Their choice is mainly driven by the hypothesis concerning the type of modification induced by the effect [60]. Batch correction must be applied carefully to avoid the removal of real biological signals. In a model training process involving dataset splitting, batch correction should be performed in each training fold to avoid information leakage.

Concerning missing data, there is a classification of the inherent mechanism that may govern the handling method: missing not at random (MNAR), missing at random (MAR) and missing completely at random (MCAR) [61]. Traditional approaches to handle missing data are based on naive data imputation, with some problems of variance reduction and bias [62]. Advances in AI and notably deep learning allow the exploration of new tools to tackle this issue, for example through the parallel integration of different omics data types [62]. The upcoming concept of the foundation model, using the attention mechanism of transformers [63], could find promising applications to more robustly handle the problem of missing data and/or modalities.

#### 2.2.3. Model Development Pipeline for Clinical Application

A robust development pipeline helps anticipate, characterize and reduce these different sources of uncertainty that might impair model outcome. Development of predictive multi-omics-based models in oncology may benefit from methodological frameworks that have already been undergone for similar tools, notably modeling and simulation in healthcare. Galappaththige and coworkers offered an overview of the credibility assessment process for patient-specific models using patient-specific cardiac electrophysiological (EP) modeling as an example [64]. They underline three main activities, verification, validation and uncertainty quantification, as well as various concepts, like context of use or risk assessment, that should be undertaken during the development of a computational model in healthcare, to ensure its credibility and its robustness.

**Figure 3:**
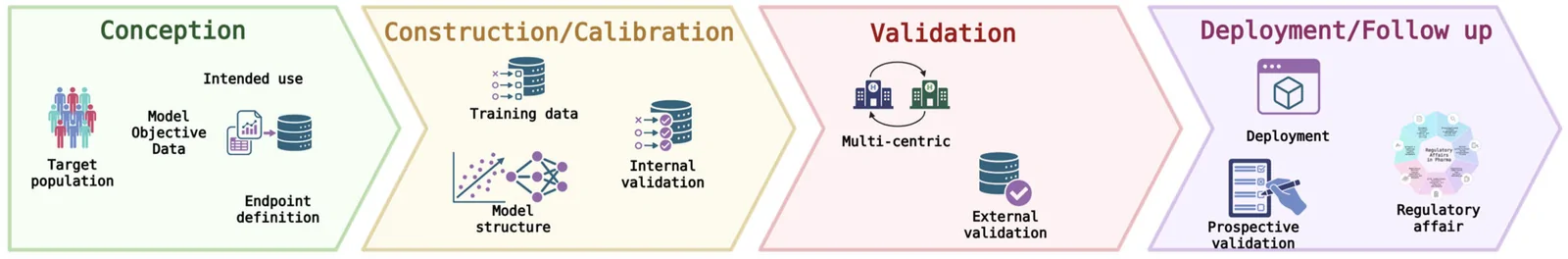
Development pipeline for omic-based predictive model for clinical application. A robustly defined pipeline permits the identification of the challenges that may arise at the different steps of model development, and of potential solutions or mitigation.

Such frameworks could be extended and adapted to the construction of omics-based predictive models. It should encompass different actions at all stages of development of the predictive model, as summarized in Figure 3: during the conception phase, a clear definition of the target population, the intended use and the endpoint helps to underline the requirement of a functional model, the strategy for risk management and the availability of resources for training. Then, during the construction and calibration phases, attention should be paid to the model structure, the feature selection and the training process. The concept of internal and cross-validation may be of use to assess good model training and avoid problems like overfitting and leakage. Then, a linked phase is the phase of validation, especially external validation, evaluating a model’s prediction capacity in new environments, its potential bias and its robustness against phenomena like center/assay effects, dataset shift or temporal drift. It can use some metrics, like decision-curve analysis [65]. The final phase is the deployment phase with monitoring of real-world model performance and possible prospective validation.

In this phase, a model can face other types of difficulties from an operational perspective: assay cost, turnaround time, tissue requirements, FFPE compatibility, analytical validity, clinical utility, regulation, reimbursement, and workflow integration [66].

#### 2.2.4. Explainability

The wider clinical adoption of predictive models will require trust in the model predictions. This trust relies on good predictions, and also on understanding how the model makes a particular prediction. This concern directly addresses the “black box” problem often highlighted with ML models [67]. The field of XAI comprises different methods to generate metrics that help us to understand how a model works, and this is beginning to be used in models for precision oncology [68,69]. In particular, XAI methods have been used in combination with several ML algorithms that predict the treatment response from multi-omics data in ovarian cancer to highlight the key determinants of the treatment response among all multi-omics variables [70]. Among these methods, the SHapley Additive exPlanations (SHAP) method [71], which assigns to each variable a contribution to the prediction by computing its average Shapley value (approximation) over all possible feature combinations, has become by far the most widely adopted attribution technique in the medical and oncology literature [72,73,74]. This popularity should not, however, be conflated with mechanistic or causal validity: SHAP primarily addresses model transparency, i.e., how a trained model weighs its inputs, and offers only an approximate, model-specific measure of attribution stability. In omics data, where features are frequently correlated (e.g., co-regulated genes, linked pathway members), SHAP values can be unstable and importance can be arbitrarily shared or shifted among correlated variables. A feature flagged by SHAP should therefore be treated as a hypothesis of biological plausibility rather than as evidence of a mechanistic role and should be confirmed through independent statistical validation (e.g., stability or permutation-based robustness checks, replication in independent cohorts) or functional validation.

## 3. Modeling

**Figure 4:**
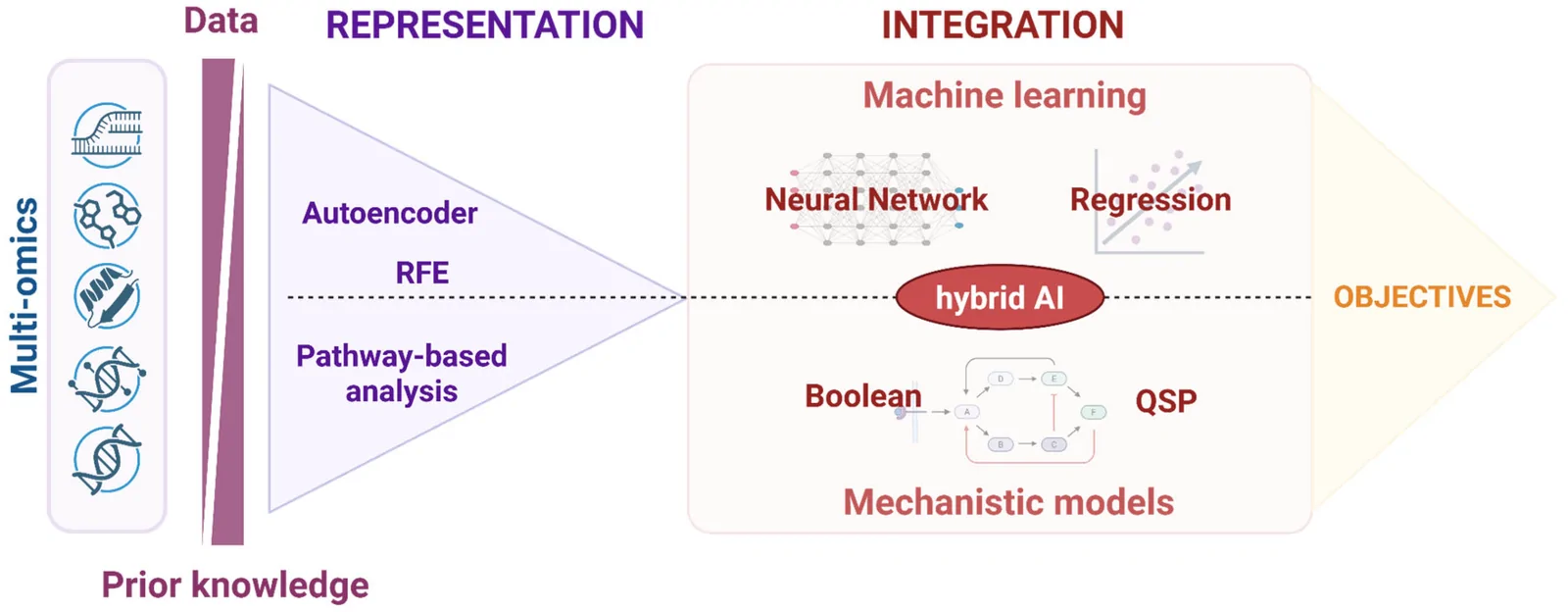
Multi-omics-based conceptual pipeline for predictive oncology. The high dimensionality of multi-omics data requires a preprocessing step that we named “Representation”, before the second step of “Integration” in a model to perform the prediction. Various techniques exist to perform the two steps, and the choice depends on the balance between the amount of data and the extent of prior knowledge relevant to the model prediction objective. RFE: recursive feature elimination, AI: artificial intelligence, QSP: quantitative systems pharmacology.

Besides a good adequacy between the available data and the prediction objective, the choice of modeling approaches is of paramount importance for achieving accurate prediction. To build a predictive model, one needs to find the best way to extract as much information as possible for the specific use case. The specific features of omics data require additional caution to avoid common traps (overfitting) when integrating them into models to perform precision oncology. We identified two major steps in a modeling pipeline that are summarized in Figure 4. We call the first step “Representation”. It relates to the best data shape to retrieve the most information for the prediction objective. The second step “Integration” is how to find the best function shape to maximize the accuracy of the link between the input and the prediction objective.

### 3.1. Representation Step

One of the major challenges in omics data analysis is the curse of dimensionality that requires careful data processing [75]. In the case of multi-omics the existing dialogue and redundancy between the different omics data bring additional considerations: notably, the timing of the integration of the different omics data type, with the notion of early, intermediate and late integration [15]. Wu and coworkers already offered an extensive review of variable selection methods for multi-omics integrations, distinguishing supervised, semi-supervised and unsupervised integrative analysis, within parallel and hierarchical integration studies [76]. In this section, we decided to give an updated overview of these different feature-selection methods based on the postprocessed data format: a subset of the initial feature set and/or feature into a transformed information space, which we believe are applicable both for single-omic- and multi-omics-based predictive models.

#### 3.1.1. Selection

In traditional ML algorithms, selection techniques, mainly Least Absolute Shrinkage and Selection Operator (LASSO) or recursive feature elimination (RFE), are directly embedded in their training process and can be applied to omics data [77]. Both techniques involve fitting an ML model to a metric of interest, while minimizing another metric (the number of variables for RFE [78] or the absolute value of model parameters for LASSO [79]) that helps to select the most relevant variable(s). However, when the training algorithm is costly or less adapted to such selection, it may be interesting to use feature selection independently from the model. Variance-based methods can offer a good initial selection filter [80]. For instance, Perception, a precision oncology computational pipeline, predicts the treatment response from patient single-cell transcriptomics data using ML models. The pipeline was built using transfer learning on data from different contexts. The authors performed feature selection using the Pearson correlation coefficient method to select the genes with a bulk expression that correlated with the in vitro viability profile of cell lines in drug screens [81]. Another problem that can arise from standard feature-selection methods is a lack of robustness and the reproducibility of selected features. Yang and coworkers proposed a robust variable-selection framework based on least-absolute-deviation LASSO and showed effective identification of genomic features on TCGA datasets [82]. Interestingly, the behavior of omics data could be used as a source of information to guide data selection. In Dandou et al., we selected genes based on their distance from the biological pathway targeted by the drug types whose efficacy we wanted to predict [47]. This selection improves the prediction performance compared with an approach that takes all available genes into account [47]. Additionally, XAI could also be used to improve feature selection. Wang and coworkers propose a selection method that considers explanations besides traditional curation and retention rate metrics. They used this method to perform binary classification of lung cancer patients based on clinical data [83].

#### 3.1.2. Transformation

In some cases, variable selection and transformation allow the emergence of inputs that are more related to the prediction objective. The best-known technique is principal component analysis (PCA), aiming to preserve the maximum input variance while reducing the number of dimensions, and has been used for predictive purposes in oncology [84]. Universal manifold approximation and projection (UMAP) is another well-known dimension reduction technique that has been preferably used with omics data [85]. Deep learning methods offer powerful new tools to better extract useful information from omics data. Huang and coworkers used neural networks to extract relevant features for Cox regression survival analyses in breast cancer [86]. Auto-encoders and variational auto-encoders are a type of neural network that project their inputs into a latent space. Their potentiality in terms of dimensionality reduction is particularly adapted to the high dimension of omics data [87,88], even if one must pay attention to the risk of overfitting in the high dimension–low sample paradigm through careful design of latent space dimension. Hira and coworkers built variational auto-encoders that compress multi-omics data into robust features to predict ovarian cancer prognosis. The more recent concept of transformers offers promising perspectives in terms of embedding, but requires a higher quantity of data [89,90]. One limitation of transformation methods is the difficulty of latent space interpretation. Different groups have tried to integrate some knowledge at different steps of the process [91,92,93], notably using the concept of graphical representation of the data [94,95]. Other methods for guided transformation are even more knowledge-based. From a function point of view, the proteins encoded by genes do not act separately, but rather in a network or in pathways. Thus, omics information can be grouped into entities, represented by a mean value obtained with gene set enrichment analysis or pathway activity inference, which can be exploited to perform prediction [31,96,97]. DrugCell is an interpretable deep learning model to predict the response of tumor cell lines to drugs [98]. It uses visible neural networks, composed of subsets of nodes that represent physiological subsystems and the connectivity of which replicates biological connections [98].

### 3.2. Integration Step

The objective of the predictive model fundamentally challenges the hypothesis regarding the relationship between inputs and outputs [99]. Depending on the used models, this hypothesis is formulated with varying degrees of clarity, and this can influence the model performance and also the transparency of its decision-making process.

Traditionally, linear regression models have been used as well as more complex models such as random forest and XGBOOST [100,101]. They rely on a quite simplistic association to link variables with the desired model outputs. When this assumption impairs model precision, deep learning models based on neural networks can offer a viable alternative because they can represent all types of functions and relationships, at the cost of a reduced interpretability. Qiao and coworkers have highlighted equivalent performances between classical machine learning and deep learning for predictive tasks in the bone tumor context (not only multi-omics), with particularities depending on the type and the structure of the data [102]. Sharifi-Noghabi and coworkers used deep neural networks to integrate somatic mutations, copy number aberrations and gene expression data to predict the drug response with improved performance compared to standard statistical methods [103].

In a knowledge-rich environment, the model structure can be derived from the known biological interactions between entities. Knowledge network is a form of structural representation of knowledge that is well adapted for biological system representation. Its use has been popularized by the creation of integration databases, such as KEGG [104]. They represent a great tool to enhance the understanding of cancer pathophysiology [105] and form the structural backbone of knowledge-based models, or “mechanistic models” [106].

Mechanistic models can be divided into different groups, depending on their consideration of time, granularity level and quantitative aspect. Boolean networks can precisely and qualitatively describe complex biochemical interaction networks and gene–protein integration [107]. Quantitative systems pharmacology models predict the temporal evolution of biological quantities using ordinary differential equations (ODEs), the rates of which integrate knowledge-derived variable dependencies [108]. Mechanistic models allow understanding and discovering pathophysiological phenomena and also promising treatment combinations. From a precision oncology perspective, it is generally difficult to directly integrate omics data in such models [11]. Yet, some research groups pursued this direction with promising results. Beal and coworkers integrated gene expression data in a knowledge-based Boolean model [109] to create a cell-line-specific model that represents the activation level of intracellular pathways [109]. They showed that it helps to understand the heterogeneous response to some targeted therapies. Similarly, Lewis and coworkers built personalized flux-based models to study the resistance to radiation therapy [110]. They used well-defined rules to derive quantitative patient-specific information from proteomic, genomic, kinetic and thermodynamic data to build a representative model of the metabolic fluxes at the genome scale. With this model, they identified differences between radiation-sensitive and radiation-resistant tumors.

All these processes require predefined rules to integrate the values derived from omics data into the model. However, this may limit the benefits of large-scale datasets and prevent the identification of relevant pathophysiological patterns. Hybrid models could allow exploiting the advantages of both, thus facilitating the discovery by ML of hypotheses that are relevant for the prediction objective in an environment constrained by the already existing knowledge. To tackle the issue of predicting tumor dynamics from early observations, Laurie and coworkers integrated an auto-encoder architecture into the classical ODE model for that problem type [111,112] to infer patient-specific kinetic parameters that turned out to be robust predictors of overall survival. The difficulty lies in finding the most adapted merging methods and the appropriate balance between existing knowledge and available data [113]. Tognetti and coworkers [114] used the protein activation dynamics in tumor cell lines from in vitro perturbation experiments to obtain cell-line-specific parameters for the mechanistic logic-ODE model of intratumoral pathways, the general structure of which was based on a prior-knowledge network. Then, they compared the predictions of the cell line response to anticancer drugs in vitro using these cell line-specific parameters against general RNA-sequencing information. Prediction was performed using the Macau algorithm, a Bayesian multitask multi-relational algorithm [115]. Interestingly, RNA-sequencing data were better predictors for a large number of drugs. Conversely, the cell-line-specific parameters permitted better prediction for drugs that targeted tumor pathways represented in the mechanistic model [114]. This showed that these parameters can capture some information about cell-line-specific dynamics of tumor pathways, relevant for drug efficacy. Then, one could hypothesize that these parameters reflect the state of intratumoral signaling pathways in the cell lines. Future studies could explore the relationship between these mechanistic parameters and the multi-omics profile of a given cell line. The lack of knowledge would make an ML model more appropriate. If predictive, this model could become the first step of a hybrid pipeline to predict cell line drug response using multi-omics data. This hypothetical project exemplifies the potential of considering both knowledge and machine meaning-based approaches in precision oncology.

## 4. Conclusions and Future Perspectives

The increasing adoption of multi-omics techniques represents a tremendous opportunity to advance precision oncology, in addition to the already widely used imaging methods. Multi-omics data are a very specific kind of data due to their high dimensionality and the inherent biological processes from which they are retrieved. Several groups have developed models that integrate omics/multi-omics data for precision oncology, also leveraging the recent improvements in the field of AI and deep learning. However, the translation of these models from the bench to the clinic still faces many challenges in terms of prediction robustness and transparency.

To overcome these obstacles, here, we discussed the necessity to understand the complexity of building a predictive model to be used in precision oncology through three key concepts: the objective, both in terms of outcome and context of the prediction application; the data, their format, variability and availability for training; and the model used for the prediction. We think that the alignment of these three concepts should result in more robust prediction models, more likely to be used in the clinic.

Cancer is a complex disease in which many involved physiological mechanisms are altered and with a strong heterogeneity. Increasing the corpus of knowledge at different scales and in many biological models has helped to draw an integrated map of the cancer hallmarks that strengthens our understanding of the disease. We think that considering this knowledge in the global picture of a precision oncology project could guide the decision-making process at different steps and allow the integration of multi-omics data to maximize the information they can provide.

Lastly, the emergence of methods that integrate entities previously considered separately (e.g., multimodal models that integrate multi-omics data with imaging or clinical data, or AI hybrid models that mix a mechanistic and ML-based architecture) could pave the way to new models that might grasp cancer complexity and might result in significant progresses in cancer management.

## 5. Material and Methods

This is a narrative review based on a critical and selective synthesis of the scientific literature on omics-based predictive models in oncology. Sources were identified through non-systematic searches of PubMed between 2016 and 2025, with particular attention to computational modeling, multi-omics integration, knowledge integration, and AI- and ML-based models, for studies published within the selected time window (last date of search: July 2026). Additional references were included through backward and forward citation tracking. Given the interdisciplinary scope and the rapidly evolving nature of the fields addressed (multi-omics and AI), this review was conceived as a narrative synthesis rather than a systematic review. Consequently, no formal PRISMA protocol or predefined inclusion/exclusion criteria were applied, which may introduce selection bias. To mitigate this limitation, the literature identification combined targeted PubMed searches within a defined temporal window (2016–2025) with backward and forward citation tracking of key articles from large consortia and high-impact studies.

Table 1 recapitulates the list of publications involving omic-based prediction models for oncological purposes integrated in the review.

## Figures and Tables

**Table 1:** Summary of the studies involving prediction models for oncological purposes integrating omic data, cited in the manuscript. Only the studies for which we could fill all the columns were kept.

Title	Cancer Type	Omics Modality	Objective	Architecture (Integration, Model)	Validation Type
Integrating Multi-Omic Data with Deep Subspace Fusion Clustering for Cancer Subtype Prediction [29]	Pancancer	mRNA expression, DNA methylation, miRNA expression (TCGA)	Cancer subtype prediction (survival analysis)	Deep Subspace Fusion Clustering (DSFC)—SNF	Cross-validation
Multiomics Integration and Machine Learning Reveal Prognostic Programmed Cell Death Signatures in Gastric Cancer [34]	Gastric cancer	Transcriptomic, epigenetic, and somatic mutation data	Cancer clustering + develop a signature for survival prediction	CoxBoost, stepwise Cox, Lasso, Ridge, elastic net (Enet), survival support vector machines (survival-SVMs), generalized boosted regression models (GBMs), supervised principal component (SuperPC), partial least squares Cox (plsRcox), and random survival forests (RSFs)	External validation
Comprehensive Multi-Omics Integration Uncovers Mitochondrial Gene Signatures for Prognosis and Personalized Therapy in Lung Adenocarcinoma [37]	Lung cancer	Transcriptomic	Cancer clustering + develop a signature for survival prediction	Ensemble of machine learning model	Cross-database
Machine Learning and Mechanistic Modeling for Prediction of Metastatic Relapse in Early-Stage Breast Cancer [38]	Breast cancer	Clinicopathologic	5-year metastatic relapse prediction	Mechanistic model + classic ML	Cross-validation
Predicting Tumor Response to Drugs Based on Gene-Expression Biomarkers of Sensitivity Learned from Cancer Cell Lines [41]	Pancancer	Transcriptomic	Drug response prediction/gene signature (from in vitro to in vivo)	GA/KNN (genetic algorithm/k-nearest neighbors) algorithm combining a genetic algorithm for feature selection and the k-nearest neighbor method for classification or prediction	In vivo prediction from in vitro data
Anticancer Drug Response Prediction Integrating Multi-Omics Pathway-Based Difference Features and Multiple Deep Learning Techniques [42]	Pancancer (cell line)	Transcriptomic	Drug response prediction	Pathway activity + drug representation	Data splitting strategy
A Gene Signature of Survival Prediction for Kidney Renal Cell Carcinoma by Multi-Omic Data Analysis [43]	Kidney renal carcinoma	Transcriptomic, methylation	Build a multi-gene prognosis signature for KIRC	LASSO Cox regression	Internal validation
Improved Prediction of MAPKi Response Duration in Melanoma Patients Using Genomic Data and Machine Learning [47]	Melanoma	Genomic + clinic	Prediction of response duration	XGBOOST + data preprocessing	Cross-validation
PERCEPTION Predicts Patient Response and Resistance to Treatment Using Single-Cell Transcriptomics of Their Tumors [81]	Myeloma, breast cancer, lung cancer	Bulk/single-cell transcriptomics	Prediction of drug response, transfer from bulk to single cell	Elastic net regression model	External
Integrate Multi-Omics Data with Biological Interaction Networks Using Multi-View Factorization AutoEncoder (MAE) [91]	Pancancer	Multi-omics	PFS prediction	Neural network (3-layer)	CV + internal
scGen Predicts Single-Cell Perturbation Responses [92]	Not only cancer	Single-cell gene expression	Perturbation prediction	Auto-encoder + latent space arithmetic	External (several datasets)
Predicting Drug Response and Synergy Using a Deep Learning Model of Human Cancer Cells [98]	Pancancer	Genomic data	Drug response prediction	Visible neural network, biological pathway integration	Cross-validation
A Machine Learning Framework Develops a DNA Replication Stress Model for Predicting Clinical Outcomes and Therapeutic Vulnerability in Primary Prostate Cancer [101]	Prostate cancer	Gene expression	Prognosis prediction	Cox regression, XGBoost	Cross-validation
MOLI: Multi-Omics Late Integration with Deep Neural Networks for Drug Response Prediction [103]	Pancancer	Gene expression, genomic	Drug response prediction	Neural network	External validation
Deciphering the Signaling Network of Breast Cancer Improves Drug Sensitivity Prediction [114]		Genomic, gene expression, proteomic	Drug response prediction	Prior-knowledge-based logic-ODE model, Macau algorithm	Translational validation (on PDX)

## Data Availability

No new data were created in this work.

## Abbreviations

The following abbreviations are used in this manuscript:

AI	Artificial intelligence
LASSO	Least Absolute Shrinkage and Selection Operator
ML	Machine learning
MAPK	Mitogen-activated protein kinase
ODE	Ordinary differential equation
PTM	Post-translational modification
QSP	Quantitative systems pharmacology
RFE	Recursive feature elimination
UMAP	Universal manifold approximation and projection
XAI	Explainable Artificial Intelligence
